# Focused Echocardiography to Guide Management in Acute Decompensated Heart Failure: From Rapid Phenotyping to Therapeutic Precision: Short title: Focused Echocardiography in the Management of Acute Decompensated Heart Failure

**DOI:** 10.31661/gmj.v15i.4224

**Published:** 2026-05-28

**Authors:** Zakieh Amiraslanzadeh, Elnaz Javanshir

**Affiliations:** ^1^ Cardiovascular Research Center, Tabriz University of Medical Sciences, Tabriz, Iran

**Keywords:** Echocardiography, Heart Failure, Therapeutic Precision, Rapid Phenotyping

## Dear Editor,

**Figure-1:**
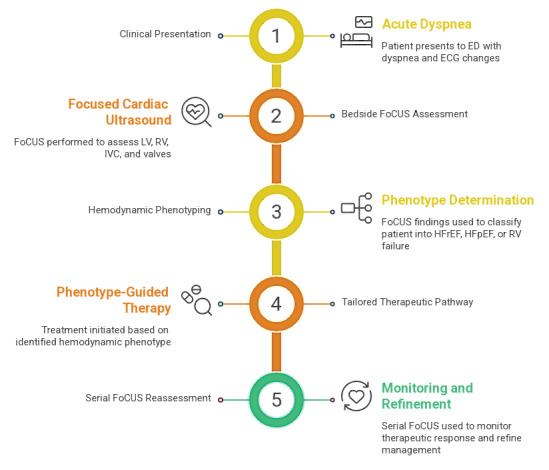
Figure 1. Proposed Algorithm for Integrating FoCUS in ADHF Management

**Table:** Table1. Clinical Utility of Focused Echocardiography in ADHF

**Clinical Question**	**FoCUS Parameter**	**Therapeutic Implication**	Reference
Is systolic function reduced?	Semi-quantitative visual LVEF estimation	Initiate or optimize guideline-directed therapy; consider inotropic support in shock	[1][19]
Is there significant right ventricular dysfunction?	RV size and qualitative systolic performance (e.g., TAPSE when feasible)	Adjust diuretic strategy; evaluate pulmonary pressures; consider advanced support	[16][17]
Is congestion present?	Inferior vena cava diameter and collapsibility	Guide intensity of diuresis and fluid management	[4][7]
Is there structural complication?	Pericardial effusion or severe valvular abnormality	Urgent cardiology consultation and comprehensive echocardiography	[1][18]

Acute decompensated heart failure (ADHF) remains a leading cause of hospitalization and readmission worldwide, with ongoing challenges in timely diagnosis, hemodynamic characterization, and individualized management [1]. Despite advances in guideline-directed therapies, early phenotyping in acute settings frequently relies on clinical examination and biomarkers, both of which have recognized limitations in accurately estimating filling pressures and volume status [2][3].

Focused echocardiography (FoCUS) has emerged as a pragmatic, bedside imaging modality capable of rapidly characterizing cardiac structure and function [4]. We highlight the evolving role of FoCUS as an adjunct to clinical assessment in ADHF, particularly in time-sensitive environments where immediate hemodynamic insight may influence early management decisions [5]. Although comprehensive echocardiography provides detailed structural and Doppler-based evaluation, it may not always be immediately available in acute care settings [6].

FoCUS, performed at the point of care, enables targeted assessment of left ventricular systolic function, right ventricular (RV) size and performance, gross valvular abnormalities, pericardial effusion, and inferior vena cava (IVC) dynamics as a surrogate marker of volume status [7]. Observational studies suggest that integration of point-of-care cardiac ultrasound with clinical examination improves diagnostic accuracy for heart failure syndromes compared with clinical evaluation alone and may refine congestion phenotyping [1]. Contemporary heart failure guidelines, including those from the American College of Cardiology and the European Society of Cardiology, increasingly acknowledge the role of bedside imaging in acute settings, particularly for differentiating cardiogenic from non-cardiogenic causes of dyspnea and supporting early therapeutic decisions [8]. In particular, European guidance has discussed ultrasound-based assessment of congestion (e.g., inferior vena cava metrics) as an adjunct to clinical evaluation, while lung ultrasound is frequently referenced for pulmonary congestion assessment [2][3]. However, these recommendations are primarily based on expert consensus and non-randomized evidence, underscoring the need for further validation[8][9].

Beyond diagnostic clarification, FoCUS may have meaningful therapeutic implications. In emergency and critical care settings, point-of-care cardiac ultrasound has been associated with changes in treatment plans, shorter time to diagnosis, and reduced downstream testing [10][11][12].

In ADHF specifically, FoCUS may facilitate earlier initiation and titration of diuretic therapy, refine decongestion strategies, and inform triage decisions. Although available evidence remains predominantly observational, findings across diverse care settings suggest that FoCUS can support structured heart failure management pathways [13][14][15].

Early semi-quantitative estimation of left ventricular ejection fraction (LVEF) allows rapid stratification into reduced versus preserved systolic function phenotypes, which may influence prioritization of pharmacologic or device-based strategies [16]. Similarly, identification of significant RV dysfunction or suspected pulmonary hypertension may prompt cautious diuretic titration, reassessment of preload-reducing therapies, or consideration of advanced support in selected patients [17]. These treatment considerations are mechanistically plausible; however, robust randomized trials demonstrating improved survival, reduced rehospitalization, or enhanced functional status with FoCUS-guided strategies remain limited.

Serial focused examinations have been proposed as a strategy to monitor response to diuretic therapy by tracking dynamic changes in ventricular filling and IVC variability, complementing clinical markers such as weight change and natriuretic peptide trends [4].

Emerging research on ultrasound-guided decongestion suggests that imaging markers of residual congestion are associated with adverse outcomes [1].

Nevertheless, such associations do not establish causality, and whether imaging-guided therapeutic adjustment improves mortality, length of stay, or readmission rates remains uncertain.

Importantly, FoCUS should not be regarded as a substitute for comprehensive echocardiography but rather as a complementary, hypothesis-driven extension of bedside assessment embedded within a structured clinical framework [18].

The technique is inherently operator-dependent, and visual LVEF estimation may exhibit inter-observer variability. Moreover, simplified protocols do not permit detailed diastolic assessment, quantitative valvular analysis, or advanced hemodynamic profiling. Standardized training, competency-based evaluation, and clearly defined scanning protocols are therefore essential to ensure reproducibility and minimize misclassification [1][19].

In table 1, we summarize the principal clinical applications of FoCUS in ADHF and their potential therapeutic implications. Figure 1 illustrates a conceptual schematic to enhance structural clarity and facilitate practical implementation within acute care pathways.

In conclusion, focused echocardiography represents a clinically meaningful extension of bedside assessment in acute decompensated heart failure. When implemented within standardized protocols and supported by appropriate training, it may enhance diagnostic precision and support individualized therapeutic strategies. However, definitive evidence demonstrating improvement in major clinical endpoints remains limited, and further high-quality randomized trials will be essential to define its true impact.

## Conflict of Interest

The authors affirm that there are no competing interests or conflicts of interest to report.

## AI Disclosure Statement

During the preparation of this manuscript, the authors used ChatGPT, OpenAI company for language editing, grammar improvement, and liboberry.com for reference management. After its use, the authors thoroughly reviewed, verified, and revised all AI-assisted content to ensure accuracy and originality. The authors take full responsibility for the integrity and final content of the published article.
